# Idiopathic hypertrophic pachymeningitis (IHP) causing headache in a young female

**DOI:** 10.4314/gmj.v59i2.8

**Published:** 2025-06

**Authors:** Smaran Korada, Viswanathan Pandurangan, Divya J Manickam, Devasena Srinivasan

**Affiliations:** Sri Ramachandra Institute of Higher Education and Research, Porur, Chennai-600116, India

**Keywords:** Pachymeningitis, Dura mater, Idiopathic, Hypertrophy, Headache

## Abstract

Hypertrophic pachymeningitis (HP) is characterised by thickening of the cranial or spinal dura mater, and it encompasses idiopathic and secondary forms. Later is linked to infections, inflammatory conditions, and autoimmune disorders. This rare condition presents with a myriad of neurological manifestations, including headaches, cranial nerve palsies, and visual disturbances. A South-Asian woman in her mid-thirties presented with a three-month history of left-sided headache, double vision, and facial numbness. Neuroimaging revealed diffuse thickening of the dura mater, suggestive of hypertrophic pachymeningitis (HP). Diagnostic workup excluded infectious and autoimmune causes, categorising the condition as idiopathic. Treatment with corticosteroids led to significant improvement in her symptoms. HP is a rare cause of headache in young patients, and diagnosis relies on neuroimaging and CSF analysis, necessitating the exclusion of secondary causes through comprehensive evaluation. Treatment varies based on the underlying aetiology. Corticosteroids remain the first-line drug for idiopathic cases.

**Funding:**

None declared

## Introduction

Pachymeningitis refers to inflammation or disease affecting the dura mater. Hypertrophic pachymeningitis (HP) involves the thickening of the cranial or spinal dura mater. This condition was first described by Charcot and Joffroy in 1869.^1^ This disease can be categorized into idiopathic and secondary forms, with secondary cases often attributed due to infections such as tuberculosis, syphilis, and fungal aetiology, autoimmune/inflammatory disorders like sarcoidosis, granulomatosis with polyangiitis, systemic lupus erythematosus (SLE), rheumatoid arthritis (RA), vasculitis, and IgG4-related diseases. Idiopathic hypertrophic pachymeningitis (IHP) is a diagnosis arrived at after ruling out secondary causes by extensive evaluation based on clinical context and presentation. Common symptoms and signs include headaches, cranial nerve palsies, visual disturbances, motor weakness, and seizures. HP is relatively rare, and reported incidence rates vary, with some case series indicating approximately 0.949 cases per million population.^2^ Currently, there are no established treatment guidelines for hypertrophic pachymeningitis. In this report, we present a case of idiopathic hypertrophic pachymeningitis, focusing on its clinical presentation, diagnostic workup, and clinical outcome.

## Case Report

A South-Asian woman in her mid-thirties, with no underlying medical comorbid illness, presented to the outpatient department with a three-month history of left-sided headache of compressive nature (pain score 6/10 on a visual analogue scale rating), each episode lasting for 6-8 hours, not associated with nausea or vomiting. The headache did not worsen on coughing or change in head position. She also reported double vision for one month and reduced sensation over the left side of her face for 15 days. The patient did not have any fever, trauma, redness of eyes, neck pain, toothache, swelling of face or eyelids. She did not have any history of weakness of limbs, involuntary movements, altered sensorium, speech disturbance, gait disturbances or urinary incontinence. She is a mother of two children, with uneventful pregnancies.

On examination, the pulse rate was 82 beats per minute, blood pressure 130/80 mmHg, and respiratory rate 18 breaths per minute, with a body mass index of 28.2 kg/m^2^. There were no external markers of tuberculosis or malignancy. Neurological examination revealed restricted abduction in the left eye, suggestive of paralysis of the left lateral rectus muscle (cranial nerve VI), and other extraocular movements were normal in the left eye. Extraocular movements were normal in the right eye. Vertical gaze in both eyes and the convergence reflex were intact.

Diminished pain, touch, and temperature sensation over the left side of the face was noted in the areas supplied by the maxillary and mandibular branches of the left trigeminal nerve (cranial nerve V). Reflexes for corneal and conjunctival responses were absent. Jaw jerk was present. Ocular examination showed no conjunctival suffusion, intact pupillary response to light, visual acuity (6/6 in both eyes), visual field testing by confrontation method, and colour vision was normal in both eyes. The motor, cerebellar, and autonomic nervous system examinations were unremarkable. Meningeal signs were absent. Other systemic examinations were normal.

Based on her symptoms, the initial provisional diagnosis was idiopathic intracranial hypertension with cranial nerve palsies (abducens and trigeminal nerve on the left side) as a false localising sign. Basic blood investigations showed neutrophilic leucocytosis (14200 white blood cells/mm^3^ and polymorphs-70%) with normal serum electrolytes, renal and liver function tests.

**Figure 1:**
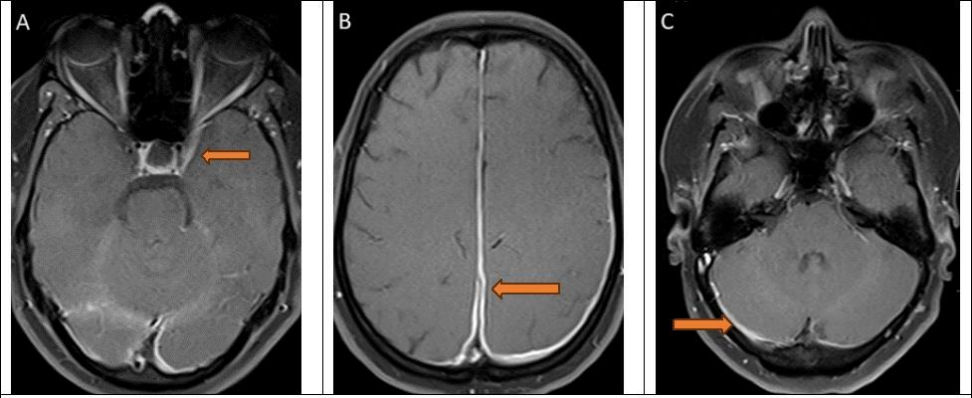
A 32-year-old woman with hypertrophic pachymeningitis presented with a headache. A) MRI Brain (axial section) shows meningeal enhancement in the left cavernous sinus extending into the left orbital apex. B) MRI Brain (axial section) shows meningeal enhancement in the interhemispheric fissure. C)MRI Brain (axial section) shows meningeal enhancement in the right cerebellar hemisphere

Acute phase reactants were elevated (erythrocyte sedimentation rate-37 mm/hr, C-reactive protein-3.8mg/dL) Chest X-ray and ultrasonogram of abdomen/pelvis were normal. Random blood sugar, glycated haemoglobin (HbA1c), urine analysis, and thyroid function tests were within normal limits. Fundus examination revealed blurred nasal margins of the optic discs bilaterally. A lumbar puncture performed showed an elevated cerebrospinal fluid opening pressure of more than 34 cm of water (normal range 6-25 cm of water). Cerebrospinal fluid analysis revealed no pleocytosis with normal sugar and protein levels and a negative screen for infectious microbiology workup, as presented in Table 1. Following the lumbar puncture, the patient did not report any worsening of headache, giddiness, or changes in sensorium. Magnetic resonance imaging (MRI) of the brain (plain and contrast-enhanced) revealed widespread thickening and inflammation of the meninges in the left parieto-occipital region, interhemispheric fissure, and right cerebellar hemisphere (Figure 1). Meningeal inflammation extended from the left cavernous sinus to the left orbital apex. These imaging findings led to a diagnosis of hypertrophic pachymeningitis.

**Table-1:** Results of cerebrospinal fluid (CSF) analysis and autoimmune/infectious diseases workup

Investigation	Patient value	Reference Range
***Biochemistry (CSF):***		
**Glucose**	65mg/dL	50-80mg/dL
**Protein**	50.8 mg/dL	>50mg/dL
**Adenosine deaminase (ADA)**	2.1 U/L	0-2.5U/L

***Cytology (CSF):***		
**Total cells**	6 cells/mm^3^	0-5cells/mm^3^
**White cell count**	5 cells/mm^3^	0-5cells/mm^3^

***Microbiology (CSF):***		
**Bacterial culture**	No growth	
**Fungal culture**	No growth	
**India ink preparation**	Not detected	
**Acid fast bacillus stain**	Negative	
**GeneXpert**	Negative	

***Autoimmune workup:***		
**ANA by Immunofluorescence**	Negative	-
**ANCA**	Negative	-
**Rheumatoid factor**	Negative	>20IU/ml
**C3**	220mg/dL	75-175mg/dL
**C4**	34mg/dL	15-45mg/dL
**Serum ACE levels**	13U/L	8-52U/L
**Serum IgG4 levels**	1.83U/L	0.03-2.01U/L

***Infectious diseases workup:***		
**HIV I Antibody**	Non-Reactive	
**HIV II Antibody**	Non-Reactive	
**HIV P24 Antigen**	Non-Reactive	
**HBsAg**	Non-Reactive	
**Anti-HCV Ab**	Non-Reactive	
**Mantoux**	Negative	
**VDRL**	Negative	

*ESR-Erythrocyte sedimentation rate; CRP-C reactive protein; ANA-Antinuclear antibodies; ANCA-antineutrophil cytoplasmic antibodies; ACE-angiotensin converting enzyme; HIV-Human immunodeficiency virus; HBs Ag-Hepatitis B surface antigen; Anti-HCV Ab- anti-Hepatitis C virus antibody; VDRL-Venereal disease research laboratory*.

Initially, the patient was started empirically on injection ceftriaxone and acyclovir intravenously, pending results of infectious workup after a discussion with a neurologist. Further extensive investigation was conducted to determine the underlying cause of the disease, which included an infectious screen and an autoimmune workup, including tests for inflammatory disorders. The comprehensive evaluation yielded negative results with no identifiable primary cause for hypertrophic pachymeningitis (Table 1).

Once the possibility of infection was ruled out, the patient was started on intravenous dexamethasone 8 mg twice daily. The patient was informed about the initiation of steroids, dysglycaemia and the need for blood glucose monitoring and possible adverse effects of steroids. The benefits of starting steroids outweigh the risks in this situation, and this was discussed in detail with the patient. Steroid was given intravenously for one week and switched to oral dexamethasone 4mg thrice a day with a weekly taper of 4mg.

Hormonal studies, which were done because of an empty sella, were normal. The patient showed significant improvement in symptoms following treatment with corticosteroids. On follow-up, the patient is responding well to the tapering regimen of oral steroids.

## Discussion

Idiopathic hypertrophic pachymeningitis is more commonly reported than secondary forms of HP. Thickening and fibrosis of the dura mater can present with a variety of clinical manifestations. This condition shows a higher incidence in men compared to women, with an average age of onset around 53 years.^3^

The possible explanations for neurological deficits seen in hypertrophic pachymeningitis include 1) mass effect exerted secondary to hypertrophied thickened dura mater, 2) fibrous tissue encasing the brain structures resulting in ischemia, and 3) congestion seen in cerebral venous sinus. The most common ubiquitous symptom is headache. Other presentations include cranial nerve palsies, cerebellar dysfunction, spinal cord involvement, seizures, visual loss, hemiparesis, radiculopathies, myopathies, and occasionally cognitive impairment, as reported in the literature.^4^ Uncommon findings in hypertrophic pachymeningitis include pituitary involvement, encephalitis, cortical venous thrombosis (CVT), and hydrocephalus.

Diffuse type involves more than 50% of the dura mater, whereas focal type involves less than 50% of the dura mater. The pattern of cranial nerve involvement correlates with the site of dural thickening, categorised into anterior and posterior patterns. In the anterior pattern, dural thickening extends from the cavernous sinus to the superior orbital fissure, resembling Tolosa-Hunt syndrome and typically resulting in cranial nerve palsies from II to VI. Conversely, the posterior pattern involves thickening of the tentorium cerebelli and posterior fossa dural thickening.

In the posterior pattern, the most common cranial nerve affected is the vestibulocochlear nerve.^5^ Virtually, any cranial nerve can be involved in IHP, except the olfactory nerve. Spinal pachymeningitis commonly affects the dura mater surrounding the cervical and thoracic spinal cord. Spinal involvement manifests as either radiculopathies or myelitis.

The aetiology of hypertrophic pachymeningitis is diverse, encompassing a range of infectious and autoimmune causes. Among infectious aetiologies, tuberculosis is the most prevalent, followed by syphilis and fungal infections like mucormycosis and aspergillosis, and Lyme disease. In middle-income and low-income countries, tuberculosis is endemic, and it commonly affects dorsal vertebrae, so it's prudent to rule out tuberculosis in pachymeningitis. Das et al., in their prospective observational study conducted among 44 patients with intracranial hypertrophic pachymeningitis, found that the cause was idiopathic in 56.8% (n = 25), and the most common secondary cause identified was tuberculosis (22.8%, n = 10). A comprehensive workup for secondary autoimmune disorders should include granulomatosis with polyangiitis, sarcoidosis, rheumatoid arthritis (RA), Sjögren syndrome, systemic lupus erythematosus (SLE), giant cell arteritis, Behçet syndrome, IgG4-related disease, and relapsing polychondritis.

The pathogenesis of idiopathic hypertrophic pachymeningitis remains unclear, characterized by inflammatory cell infiltration and interstitial fibrosis of the dura mater. According to a study by Xu Zhang et al., elevated levels of interleukin-4, interleukin-5, interleukin-9, interleukin-10, tumour necrosis factor-alpha, and vascular endothelial growth factor were observed.^7^ Notably, these interleukins belong to the Type-2 helper cell (TH2) cytokine group, suggesting a predominant involvement of TH2 cells in IHP. Interleukin-4 stimulates fibroblast proliferation, resulting in fibrosis. Another fibrogenic cytokine involved in the disease process is TGF-beta-1.^7^

Brain imaging plays a vital role in potentially diagnosing hypertrophic pachymeningitis. An MRI brain scan with contrast helps identify thickening and enhancement, as well as the site and extent of involvement of the dura mater. IHP characteristically presents with either smooth or nodular thickening, which is iso-hypointense on both T1 and T2-weighted sequences.^8^ Post contrast enhancement is attributable to fibrosis and necrosis of the dura mater. Warittikoon and Jakchairoongrauang, in their study, described MRI findings more often suggestive of IHP as T2 hypointensity with post-contrast enhancement of the dural edge.^9^

The gold standard for definitive diagnosis remains a biopsy of the dural thickening sites, providing histopathological confirmation. Pathological characteristics encompass thickening, fibrosis, and the presence of inflammatory cells such as plasma cells and lymphocytes. The presence of granulomas or vasculitis aids in diagnosis. This comprehensive approach helps in excluding secondary causes and arriving at a diagnosis of idiopathic hypertrophic pachymeningitis 10

Treatment strategies differ based on the underlying cause, which can be infectious or autoimmune-associated hypertrophic pachymeningitis. Initial management of IHP typically involves corticosteroid therapy. If the condition proves refractory to steroids, initiation of immunosuppressive agents is recommended. Surgical intervention may be considered if symptoms persist despite medical treatment. Due to the tendency for relapse, many patients require long-term maintenance therapy.^11^ Approximately 50% of patients with HP experience a relapse after treatment, with recurrences occurring anywhere from one week to several years later.^12^ The higher relapse rate advocates the need for regular follow up and monitoring in these patients. This case contributes to the limited epidemiological data. It reinforces the importance of considering IHP in patients with chronic headache with false localising signs, advocating for a multidisciplinary approach in diagnosis and treatment.

## Conclusion

Idiopathic hypertrophic pachymeningitis (IHP) is a rare and complex neurological disorder characterized by the thickening of the cranial or spinal dura mater. Diagnosis is challenging and requires an extensive evaluation to exclude infectious and autoimmune causes, with brain imaging and cerebrospinal fluid (CSF) analysis being crucial in establishing a diagnosis. Unexplained chronic headache needs workup for IHP. Treatment typically involves corticosteroids, with immunosuppressive agents reserved for cases that are refractory to corticosteroids.
